# Physical activity in US Blacks: a systematic review and critical examination of self-report instruments

**DOI:** 10.1186/1479-5868-7-73

**Published:** 2010-10-08

**Authors:** Kathleen Y Wolin, Casey Fagin, Nneka Ufere, Hallie Tuchman, Gary G Bennett

**Affiliations:** 1 Washington University School of Medicine, St. Louis, MO 63110 USA; 2 Duke University, Durham, NC USA

## Abstract

**Background:**

Physical activity self-report instruments in the US have largely been developed for and validated in White samples. Despite calls to validate existing instruments in more diverse samples, relatively few instruments have been validated in US Blacks. Emerging evidence suggests that these instruments may have differential validity in Black populations.

**Purpose:**

This report reviews and evaluates the validity and reliability of self-reported measures of physical activity in Blacks and makes recommendations for future directions.

**Methods:**

A systematic literature review was conducted to identify published reports with construct or criterion validity evaluated in samples that included Blacks. Studies that reported results separately for Blacks were examined.

**Results:**

The review identified 10 instruments validated in nine manuscripts. Criterion validity correlations tended to be low to moderate. No study has compared the validity of multiple instruments in a single sample of Blacks.

**Conclusion:**

There is a need for efforts validating self-report physical activity instruments in Blacks, particularly those evaluating the relative validity of instruments in a single sample.

## Background

Most self-report measures of physical activity in the US have be developed and validated in exclusively or largely White samples [1]. There has been limited uptake of recent calls for physical activity instruments to be validated in more racially/ethnically diverse samples [1]. There are several reasons to believe that physical activity measures may perform differently among some US Black populations.

There is some evidence that preferred recreational physical activities differ by race/ethnicity [2,3]. It is unclear whether the existing body of self-report measures, which weren't necessarily designed with diverse populations in mind, includes example physical activities that are relevant or salient for Blacks. Thus, there is reason to expect that the validity of these instruments may be lower in Blacks because the sample activities (e.g. golf, tennis) don't match the most prevalent activities (e.g. dancing[4], household activities[5]). However, this question remains largely unexamined in the empirical literature.

Barriers to physical activity and cultural perceptions of the meaning of physical activity may also vary by race/ethnicity. Qualitative research on perceptions of and barriers to physical activity suggest Blacks may conceptualize the meaning of physical activity differently from researchers [6-9]. For example, Airhehenbuwa and colleagues report most Blacks in their study believed work and daily activities to be a form of "exercise" [6]. Thus, conceptions of what constitutes physical activity may vary by race/ethnicity and may shape both how people respond and what they believe is a socially desirable response on self report.

There is clear, but unexplained discordance in physical activity measured by different assessment modes. Self report instruments from national surveys have indicated a disparity in physical activity levels between Whites and Blacks that is not apparent when objective measures are used. For example, using the National Health and Nutrition Examination Survey (NHANES) accelerometer data, Troiano et al found Black adult women accumulated 20.0 min/day of moderate or vigorous intensity physical activity, while White adult women accumulated 19.7 min/day of moderate or vigorous intensity physical activity [10]. Black and White men recorded higher levels than women that were similar to each other (37.9 and 34.6 min/day respectively). In contrast, in earlier self report NHANES data over 40% of Black women reported no recreational physical activity as compared to approximately 20% of White women [11]. Similarly, in self report data from the Behavioral Risk Factor Surveillance System (BRFSS), 46% of Black men and 53% of White men reported engaging in moderate intensity activity at least 30 minutes/day, 5 days/week or vigorous activity 20 minutes/day, 3 days/week, a difference that was statistically significant [12]. In the BRFSS, 50% of White women reported being active, but only 36% of Black women did [12]. The BRFSS data is used for the national Morbidity and Mortality Weekly Reports, which reports on the state of public health in the United States and thus, may shape professional and public perceptions of health disparities. Further, and perhaps more concerning, many measures in the literature have a psychometric structure similar to the BRFFS and NHANES instruments, which suggests a greater need for the validation of measures in Black samples. One additional possible interpretation of the disconnect between the self-reported and accelerometer measured data is the need to measure all domains of physical activity in self-report instruments. Objective measures, like accelerometry measure movement across all domains, while self-report instruments may implicitly or explicitly focus on only some types or domains of physical activity.

Physical activity instruments that focus solely on recreational or leisure physical activity may miss important domains in total physical activity, which could have significant impact on health outcomes. Many self-report instruments fail to capture occupational and household physical activity, which may represent a larger proportion of total physical activity in some sub-populations. Evenson and colleagues found that occupational activity reports were highest among Blacks and recreational activity highest among Whites, concluding studies relying solely on recreational activity may miss important data [13]. Similarly, He and Baker reported that Black men and women reported more strenuous household and occupational physical activity than White men and women [14]. In sum, there are several compelling reasons why physical activity self-report instruments may perform differently in Blacks. Given that the validity of self-report instruments generally ranges from low to moderate[15] with indications that most substantially overestimate physical activity,[1] the possibility of impaired validity among Blacks raises concerns. This report reviews and evaluates the validity and reliability of self-reported measures of physical activity in Blacks. The review also examines whether assessing multiple domains of physical activity improves validity and whether differential validity exists for Black vs. White populations.

## Methods

We conducted a systematic literature review to identify publications reporting on the validity of self-report physical activity instruments in samples of Blacks/African Americans. We searched PubMed, PsychInfo, Google Scholar, and CINAHL for reports on the validity of physical activity questionnaires published through February 2010 using the following search terms: physical activity, exercise, questionnaire, survey, self-report, validation studies, validity, socioeconomic status, and reliability. We also gathered manuscripts from the reference lists of searched reports. We then limited the review to self-report instruments that had been validated in an adult sample (age 18 and older) with at least 20% representation of self identified Blacks or African Americans. This search yielded 14 manuscripts validating 15 different instruments. We limited the final sample to studies that were all Black or reported results separately for Blacks if more than one racial/ethnic group was included. This resulted in the inclusion of 10 instruments from nine manuscripts (Table 1). In studies where the reliability of the instrument was also evaluated, we also include that information. We reviewed the reports for validity, sample size and content, and comparison measure. We included both criterion validity against a second instrument considered to be of superior quality (i.e., a gold standard) and construct validity against a related construct (e.g., blood pressure). We reviewed instrument item content as pertaining to recreational (including leisure, sport or purposeful physical activity), occupational (i.e. work), transportation, and household (including yardwork, caretaking and domestic cleaning and maintenance activities) domains.

**Table 1:** Reliability and Validity of Self-Report Physical Activity Instruments in Blacks

Name of measure	Reference	Evaluation of instrument sample	Comparison for validity	Reliability	Validity	Domains	Number of items	Time frame for recall
CHAMPS	Resnicow 2003^12^	n = 133, 78% female, all Black mean age 41	criterion validity: fitness; construct validity: BMI and blood pressure		correlation with fitness measured by VO_2max _r = 0.17 (overall), r = 0.42 (for men), r = 0.07 (for women). Correlation with BMI r = 0.02. Correlation with systolic blood pressure r = -0.02	recreational, household, transportation	41	typical week in past month
Yale Physical Activity Survey	Young 2001^13^	n = 59, 79% female, 45% Black, age 60-80	criterion validity: 7 day PAR, fitness; construct validity: BMI		Correlation with 7 day PAR r = 0.42 (Black only).	occupational, recreational	9 questions with 31 items	typical week in past month
	Resnicow 2003^12^	n = 133, 78% female, all Black mean age 41	criterion validity: fitness; construct validity: BMI, cholesterol and blood pressure		Correlation with fitness as measured by VO_2max _r = 0.12. Correlation with systolic blood pressure r = 0.02. Correlation with BMI r = 0.11.			
Seventh Day Adventist	Singh 2001^14^	n = 165, 72% women, all Black, mean age 50, majority reported education ≥ 12 years.	criterion validity: 7 day PAR, fitness, pedometer measured steps per day	r = 0.64 for women, r = 0.78 for men	Correlation with average of two 7 day PAR r = 0.65 for women, r = 0.51 for men. Correlation with pedometer r = 0.24 for women, 0.14 for men. Correlation for fitness r = -0.09 for women, r = 0.23 for men	recreational	19	usual day
Nurses' Health Study	Wolf 1994^17^	n = 235 women, 36% Black (n = 84), age 25-42	criterion validity: past week recall and diaries	Among representative sample: r = 0.59; Among Blacks only: r = 0.39	Among Blacks only, correlation with average of four past week recall r = 0.83 (deattenuated), correlation with diaries r = 0.63 (deattenuated).	recreational	8	time per week in past year
IPAQ	Wolin 2008^18^	n = 142, 64% female, all Black, age 24-67, low income population	criterion validity: accelerometer		1 minute bout: r = 0.36 overall, r = 0.58 men, r = 0.21 women; 10 minute bout: r = 0.26 overall, r = 0.48 men, r = 0.07 women	occupational, household, recreational, transportation	10	past 7 days
Paffenbarger Physical Activity Questionnaire walking items	Resnicow 2003^12^	n = 133, 78% female, all Black, mean age 41	criterion validity: fitness; construct validity: BMI and blood pressure		Correlation with fitness measured by VO_2max _r = 0.11. Correlation with BMI r = 0.04. Correlation with systolic blood pressure r = 0.05	recreational	8	past year
Study of Women's Health Across the Nation (SWAN)	Sternfeld 2000^26^	n = 13,621 women, 27% Black, age 40-55, most employed, 66% with education > high school	construct validity: BMI		POR = 1.07 for physical activity and BMI in Blacks less active than peers only. POR = 0.95 (0.94-0.97) in Blacks more active than peers.	household, transportation	19	past year
Black Women's Health Study	Carter-Nolan 2006^20^	n = 101 Black women, age 21-69	criterion validity: accelerometer, seven day diary; construct validity: systolic blood pressure		Correlation with accelerometer r = 0.28. Correlation with diary r = 0.32. Correlation with systolic blood pressure r = -0.02.	recreational	9	past year
Health Professionals Follow Up Study	Chasan-Taber 1996^19^	n = 238 men, 45% Black, age 40-75	criterion validity: past week recall, diaries	correlation of all 10 activities on instrument r = 0.41	Correlation of vigorous activity and diary among Black men r = 0.47 (crude)	recreational, occupational	15	time per week in past year
Jackson Heart Study	Smitherman 2009	reliability sample: n = 40 Blacks, 50% female, mean age 54; accelerometer sample: n = 404 Blacks, 69% female, mean age 57; pedometer sample: n = 294 Blacks, 62% female, mean age 60; mean education = 15 years	criterion validity: pedometer, accelerometer	ICC = 0.99	Correlation with accelerometer r = 0.24. Correlation with pedometer r = 0.32	recreational, occupational, household	40	past year

BMI - body mass index, CARDIA - Coronary Artery Disease in Young Adults, CHAMPS - Community Healthy Activities Model Program for Seniors, IPAQ - International Physical Activity Questionnaire, PAR - Physical Activity Recall, POR - prevalence odds ratio

## Results

Criterion validity was available for eight instruments (Community Healthy Activities Model Program for Seniors (CHAMPS)[16], Yale Physical Activity Survey[16,17], Seventh Day Adventist [18], Nurses' Health Study[19], International Physical Activity Questionnaire (IPAQ)[20], Health Professionals Follow Up Study[21], Paffenbarger Physical Activity Questionnaire[16], Jackson Heart Study[22] and the Black Women's Health Study[23]). Of these, two reports used cardiorespiratory fitness (e.g., VO_2max_)[16,18], four used objective devices (i.e., accelerometers or pedometers)[18,20,22,23], and five used a physical activity diary or more detailed physical activity recall as the criterion measure [17-19,21,23]. Samples varied in size from 59 to 404. Only four studies included information on the socioeconomic status of the study sample [18,20,22,24].

In general, criterion validity correlations tended to be low to moderate, ranging from 0.12 to 0.83. The highest correlations were reported between the Nurses' Health Study instrument and the average of four weekly recalls (r = 0.83)[19], the Seventh Day Adventist instrument and the average of two physical activity recalls (r = 0.65 in women, r = 0.51 in men)[18], the Health Professionals Follow Up Study[21] and Nurses' Health Study [19] instruments correlating with physical activity diaries (r for both = 0.63) and the Yale Physical Activity Survey correlating with a seven-day physical activity recall (r = 0.42)[17]. With the exception of the Yale instrument, which measures occupational, recreational and household activities, these instruments measure only recreational physical activity.

For self-report instruments correlated against fitness, the highest correlation was for the CHAMPS (r = 0.17) against VO_2max _[16]. The highest correlations against an objective device were the IPAQ[20], which was moderately correlated with accelerometer using a one-minute bout (r = 0.36), the Jackson Heart Study instrument[22], which was moderately correlated with pedometer-measured steps (r = 0.32) and accelerometer (r = 0.24) and the Black Women's Health Study[23], which had a moderate correlation with accelerometry (r = 0.28). The correlation with accelerometry was lower when the IPAQ was evaluated using a 10-minute bout length and among women [20].

Construct validity data was available for one instrument that did not also have criterion validity data. Construct validity measures we considered include physical function (i.e. activities of daily living), body mass index (BMI), and blood pressure. The Study of Women's Health Across the Nation (SWAN) used BMI for construct validity [24]. Correlations with physical activity were high.

Reliability was assessed for four instruments and correlations were generally moderate ranging from 0.39 for the Nurses' Health Study instrument[19] assessed two years apart to 0.78 among men for the Seventh Day Adventist instrument[18] delivered six weeks apart. The Jackson Heart Study had an ICC of 0.99 for administrations two weeks apart [22].

Three instruments (Yale Physical Activity Survey[17], Nurses' Health Study[19] and Health Professionals Follow-up Study[21]) included both Blacks and Whites in the same sample and reported the validity of the instrument separately for each. Correlations were higher for Black women (r = 0.63) than White women (r = 0.56) for the Nurses' Health Study instrument[19], but were lower among Black men (r = 0.47) than White men (r = 0.58) for the Health Professional's Follow-Up study[21]. In the largely female Yale Physical Activity Survey sample, the correlation was stronger among Blacks (r = 0.42) than among non Blacks (r = 0.30) [17]. No study directly compared the validity of multiple instruments in a Black sample as has been done for high socioeconomic status Whites[25] and older adults[26].

## Discussion

Few studies have evaluated the validity of physical activity instruments among Blacks. Of the 25 self-report instruments for adults included in a 1997 review[27], only two[16,17] have been validated in Blacks. While several instruments that have been validated since this review have been validated in Blacks (9 total instruments have now been validated in Blacks), a substantial gap remains with numerous instruments lacking validation in samples of Black adults. As a result, there is little evidence either supporting or negating the validity of self-report instruments for Black populations.

Three of the instruments (Black Women's Health Study, Jackson Heart Study, IPAQ) reported moderate correlations with accelerometer-measured physical activity. None of these has been validated in a single sample that included Blacks and Whites for direct comparison, though both the IPAQ and the Black Women's Health Study instrument (which, as noted, is nearly identical to that used in the Nurses' Health Study) have been validated in Whites in other studies. Only the IPAQ has been validated in a lower socioeconomic status, predominately Black population. Thus, given the available data, there is good evidence for recommending semi-quantitative questionnaires like those used in the Black Women's Health Study (which was an adaptation of that used in the Nurses' Health Study and Health Professionals Follow-up Study), particularly when a shorter assessment format is desired. While also relatively brief at 10 items, the IPAQ performed well and can only be recommended among men, but not women. The more detailed and lengthy Jackson Heart Study instrument also performed well. The extent to which these measures can reliably assess change in physical activity among Black samples remains largely unexplored.

We hypothesized that instruments may perform differently in Blacks because the sample activities may not be salient or the instruments may not capture the relevant domains. In fact the data suggested including multiple domains did not improve validity, the instruments with the highest criterion validity (Nurses' Health Study, Seventh Day Adventist) measure only recreational physical activity. More detailed interviewer administered instruments like the IPAQ and Jackson Heart Study measure across domains with similar moderate criterion validity scores. Thus, the presence of more items or examples and domains did not result in higher validity scores.

The lack of brief instruments to validly measure physical activity in Blacks constitutes a significant gap. The Black Women's Health Study instrument contains nine items, but the Jackson Heart Study includes 40. None of the studies evaluated the ability of the self-report instrument to validly measure change in physical activity, as would be needed in an intervention setting, marking another major gap in the literature. Few studies evaluated the reliability of the self-report instruments, highlighting another area for future research. In addition, to evaluate the ability of self-report instruments to accurately measure physical activity in Blacks on a population level, validation sample populations need to also be diverse across socioeconomic status - something that has not been well demonstrated (or well documented) in many samples to date. Wolin et al specifically recruited low socioeconomic status Blacks and found moderate correlation between the IPAQ using a one minute bout length and accelerometry, though correlations were lower among women and when using a longer bout length [20]. The samples in the Nurses' Health Study and Health Professionals Follow-up Study are of a high socioeconomic status [19,21]. Thus, the example activities listed may have been more salient, independent of race, than they would be for a lower socioeconomic Black sample. These two instruments both identified the salient activities based on those that explained the most variation in a separate sample of high socioeconomic status individuals, the College Alumni Study[28]. In contrast, Rundle et al found low construct validity for an instrument derived from the Harvard Alumni Survey, which also builds on the same source information, in a low socioeconomic status multiethnic urban population [29]. This further highlights the need to consider both race/ethnicity and socioeconomic status when evaluating the appropriateness of a self-report instrument. It also suggests that the validity of existing measures may be enhanced by tailoring the example behaviors to better match the target population, as has been done for some instruments when tailoring for international populations [30].

An additional gap in the literature on self-report instrument validity worth noting is the limited number of validations against objective measures of energy expenditure. The highest validation correlations among Blacks were for instruments validated against more detailed self-report instruments, like diaries. Given that errors between two self-report instruments may be correlated, it is not surprising that correlations are lower when comparing instruments to non self report measures such as fitness or accelerometer measured physical activity. However, despite this, moderate correlations were found for the IPAQ[20], Black Women's Health Study [23] and Jackson Heart Study[22] instruments with objective monitoring devices such as accelerometers.

The logistical and financial constraints of many community-based and large-scale observational studies make measuring physical activity objectively not feasible, particularly when physical activity is not the sole outcome. Thus, valid self-report instruments are necessary. While the unit costs of objective measure tools ($300 to 600) are declining, the associated costs of implementation remain high, as significant staff resources are necessary to distribute, collect, process, and analyze these data. In longitudinal studies with repeated measures, these costs are often much higher. Many studies for which physical activity is not the primary endpoint will likely contain these costs by relying on self report. Furthermore, self report instruments allow physical activity recommendation messages to be issued in terms that are understandable to the public, unlike those that may be based on step-counts or metabolic equivalents derived from objective devices. Thus, self-report measures of physical activity are likely to remain an important research tool for the foreseeable future.

As this review makes clear, greater attention needs to be paid to the population specific validity of self-report instruments. This point as it applies to Hispanic populations was well detailed in a recent review by Martinez et al. [31]. As we detailed above, the wide-scale use of objective devices to measure physical activity is not feasible in many investigations. However, the marginal increased cost associated with including a small validation study of a self-report instrument in samples that include a larger percentage of Blacks is certainly supported by our findings.

## Conclusion

A number of well-known physical activity self-report instruments have been validated in Black samples. However, similar to validations in whites, the validity of these instruments as compared to objective or more detailed self-reports remains modest. Contrary to hypothesis, the validity of instruments was generally similar in Blacks and Whites in the same sample. Yet, many well-known and commonly used self-report instruments that were included in a previous review remain unvalidated in Blacks further research is needed to validate physical activity measures used in intervention and epidemiologic studies.

## Competing interests

The authors declare that they have no competing interests.

## Authors' contributions

All authors read and approved the final manuscript. KYW conceived of the study, contributed to the literature review, led the writing of the manuscript. CF conducted the literature review and led the abstraction of study data. NU and HT contributed to the literature review and data abstraction. GGB contributed to the study conceptualization and manuscript preparation and provided critical editorial input to the interpretation of the data.
